# Benzyl butyl phthalate promotes breast cancer stem cell expansion via SPHK1/S1P/S1PR3 signaling

**DOI:** 10.18632/oncotarget.9007

**Published:** 2016-04-26

**Authors:** Yu-Chih Wang, Cheng-Fang Tsai, Hsiao-Li Chuang, Yi-Chih Chang, Hung-Sheng Chen, Jau-Nan Lee, Eing-Mei Tsai

**Affiliations:** ^1^ Department of Obstetrics and Gynecology, Kaohsiung Medical University Hospital, Kaohsiung 807, Taiwan; ^2^ Graduate Institute of Medicine, College of Medicine, Kaohsiung Medical University, Kaohsiung 807, Taiwan; ^3^ National Applied Research Laboratories, National Laboratory Animal Center, Nangang, Taipei 11529, Taiwan; ^4^ Department of Medical Laboratory Science and Biotechnology, China Medical University, Taichung 40402, Taiwan

**Keywords:** aryl hydrocarbon receptor, sphingosine kinase 1, sphingosine 1-phosphate, sphingosine-1-phosphate receptor 3, breast cancer stem cells

## Abstract

Understanding the regulatory mechanisms unique to breast cancer stem cells (BCSCs) is required to control breast cancer metastasis. We found that phthalates promote BCSCs in human breast cancer cell cultures and xenograft tumors. A toxic phthalate, benzyl butyl phthalate (BBP), activated aryl hydrocarbon receptor in breast cancer cells to stimulate sphingosine kinase 1 (SPHK1)/sphingosine 1-phosphate (S1P)/sphingosine-1-phosphate receptor 3 (S1PR3) signaling and enhance formation of metastasis-initiating BCSCs. BBP induced histone modifications in *S1PR3* in side population (SP) cells, but not in non-SP cells. SPHK1 or S1PR3 knockdown in breast cancer cells effectively reduced tumor growth and lung metastasis *in vivo*. Our findings suggest S1PR3 is a determinant of phthalate-driven breast cancer metastasis and a possible therapeutic target for regulating BCSC populations. Furthermore, the association between breast carcinogenesis and environmental pollutants has important implications for public health.

## INTRODUCTION

Epidemiological studies have revealed a positive correlation between phthalate exposure and both human reproductive defects and breast cancer incidence [1]. Phthalate exposure has been shown to induce proliferation, migration, invasion and tumor formation in breast cancer cells [2]. However, the mechanism by which phthalates promote breast cancer is still poorly understood.

Breast cancer metastasis is tightly correlated with patient mortality and poor clinical outcome. The presence of breast cancer stem cells (BCSCs, also called stem-like cells or tumor-initiating cells) [3] contributes to metastasis. Although they represent only a small percentage of the whole tumor, BCSCs have been isolated from diverse tumors and established cell lines based on cell surface marker expression [4], aldehyde dehydrogenase activity [5], side population (SP) assay [6] and sphere-forming ability [7]. The SP assay relies on Hoechst 33342 efflux based on the presence of ATP-binding cassette (ABC) transporters, predominantly sub-family G, member 2 (ABCG2) [4, 8].

BCSCs share molecular characteristics with embryonic and normal adult stem cells. The molecular mechanisms that regulate BCSC populations via self-renewal signals are mediated by several signaling pathways, such as chemokine (C-X-C motif) receptor 4 (CXCR4)/aryl hydrocarbon receptor (AHR) [9], sphingosine-1-phosphate/sphingosine-1-phosphate receptor 3 (S1P/S1PR3) [5] and fibroblast growth factor receptor signaling [10].

AHR is a ligand-activated transcription factor that mediates environmental toxins such as 2,3, 7,8-tetracholorodibenzo-*p*-dioxin (TCDD) and butyl benzyl phthalate (BBP) [11]. Ligand activation triggers nuclear translocation and dimerization with AHR nuclear translocator (ARNT). The activated AHR/ARNT complex binds xenobiotic-response elements (XREs) of AHR-regulated genes and modulates their expression. AHR also regulates various stem cell lineages [9, 12], and promotes BCSC populations in tamoxifen-resistant breast cancer cells but not in susceptible cells [9]. These reports suggest a possible role for AHR in generating chemotherapy-resistant metastatic BCSCs.

Bioactive lysosphingolipid sphingosine-1-phosphate (S1P) regulates cell proliferation, apoptosis, differentiation, drug resistance, invasion and migration [13, 14] in a manner dependent on the S1P receptor (S1PR), which includes the S1PR1–5 subtypes. Sphingosine originates from ceramide and produces S1P via sphingosine kinases (SPHK1 and SPHK2) [15]. S1P/S1PR activates a variety of intracellular G-protein signaling pathways [13, 16]. In the current study, we found that phthalates promote BCSCs in human breast cancer cell cultures and xenograft tumors [2].

## RESULTS

### BBP stimulation induces expansion of breast cancer SP cells

**Figure 1:**
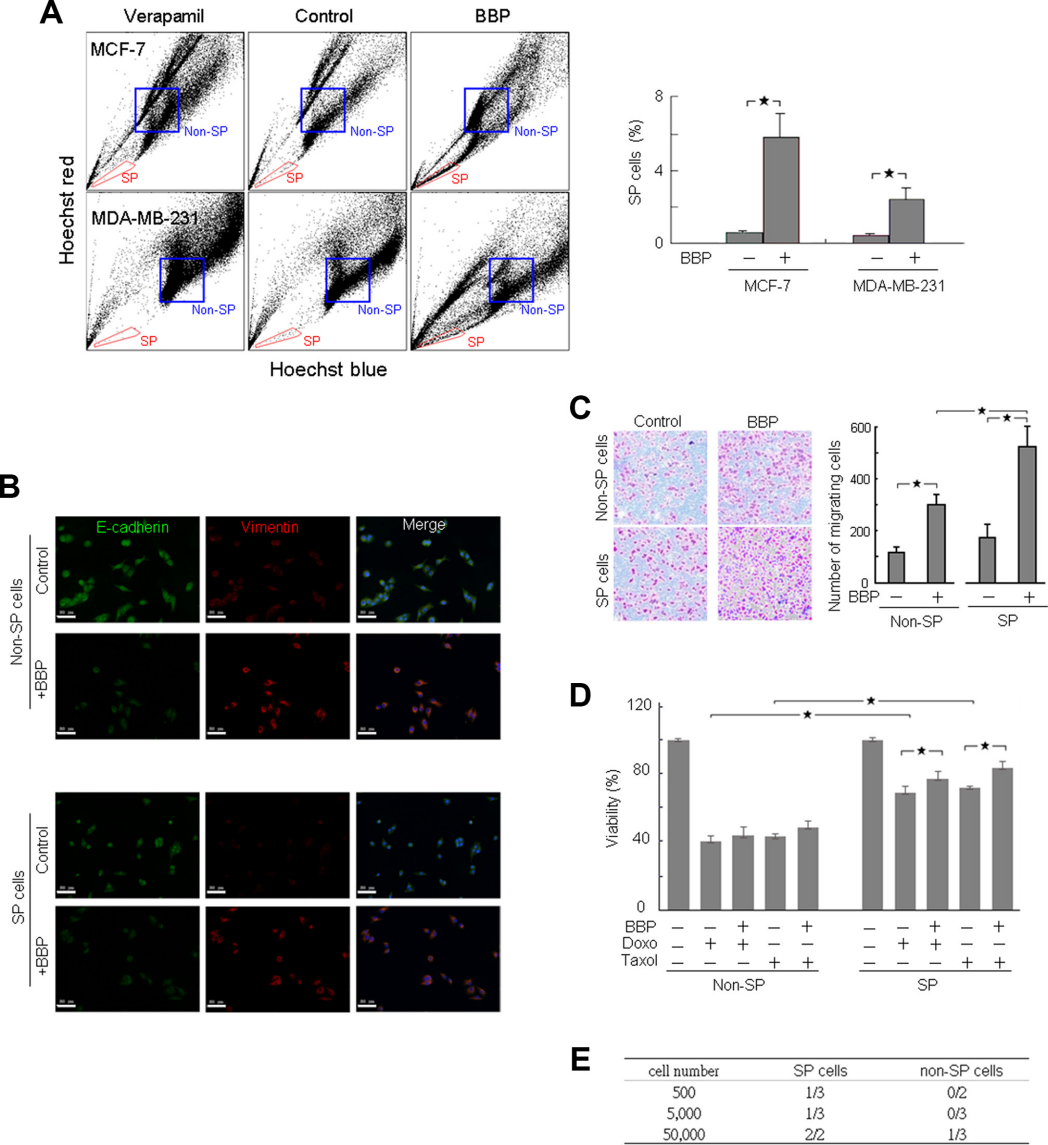
BBP significantly increased SP cell populations and promoted EMT and tumorigenicity in breast cancer cells Hoechst 33342 stained MCF-7 and MDA-MB-231 cells can be separated into SP and non-SP cells (left) (**A**) SP cells (red boxed region) were gated based on verapamil (50 μM) sensitivity (*n* = 3). FACS-sorted SP and non-SP MCF-7 cells were fixed and stained for E-cadherin and vimentin (**B**) Scale bars = 50 μm. FACS-sorted SP and non-SP MCF-7 cell migration was analyzed using the transwell assay (*n* = 3) (**C**) SP and non-SP MCF-7 cells were assessed for cell viability 24 h after doxorubicin (Doxo; 1 μM) or Taxol (5 μM) treatment with or without BBP (1 μM) (*n* = 4) (**D**) Frequency of SP and non-SP MCF-7 cell tumor formation 8–10 weeks after transplantation into nude mice, as shown by dilution experiments (**E**) Data are presented as mean ± SD; *P < 0.05.

MCF-7 and MDA-MB-231 cells were treated with 1 μM BBP or ethanol (vehicle control) for one day and the proportion of SP cells was evaluated by Hoechst efflux assay. SP cells were defined as cells that showed higher Hoechst dye efflux relative to the main population and disappeared in the presence of the ABC transporter inhibitor, verapamil. After BBP stimulation, the proportion of SP cells increased in both MCF-7 and MDA-MB-231 cells (Figure 1A). The non-SP cells were isolated as a cell population that was strongly stained with Hoechst dye.

We isolated SP and non-SP MCF-7 cells using fluorescence-activated cell sorting (FACS) to further characterize BCSCs. We previously reported that phthalate induced the epithelial–mesenchymal transition (EMT) and enhanced invasion in breast cancer cells [2]. To evaluate the effect of BBP on EMT, SP and non-SP cancer cells were initially evaluated by immunofluorescence (IF) for expression of the epithelial protein E-cadherin and the mesenchymal protein vimentin. BBP decreased E-cadherin and increased vimentin in both SP and non-SP cells (Figure 1B), suggesting that both cell types underwent EMT after BBP treatment. Transwell migration assay results showed no difference in migration activity between SP and non-SP cells in the absence of BBP (Figure 1C). BBP stimulated more cell movement in BBP-treated SP cells (3.1-fold) than in non-SP cells (2.6-fold, *P* < 0.05; Figure 1C). Following BBP treatment, SP cells were more chemoresistant than non-SP cells to common breast cancer therapy agents (doxorubicin and Taxol (paclitaxel)) (Figure 1D). BBP increased SP cell survival in the presence of cytotoxic drugs.

We evaluated the *in vivo* tumorigenic potential of SP and non-SP MCF-7 cells after subcutaneous injection into nude mice via limiting dilution transplantation. We measured xenograft formation using the Xenogen *IVIS* live imager (Caliper Life Sciences) and identified SP MCF-7 cells labeled with enhanced green fluorescent protein (EGFP). SP cells induced tumor formation more frequently than non-SP cells, particularly at low numbers of injected cells (Figure 1E). Thus, BBP-induced expansion of SP breast cancer cells appeared to increase BCSC and tumorigenic phenotypes *in vivo*.

### AHR/SPHK1 signaling induces S1P synthesis and release to promote SP cell expansion

**Figure 2:**
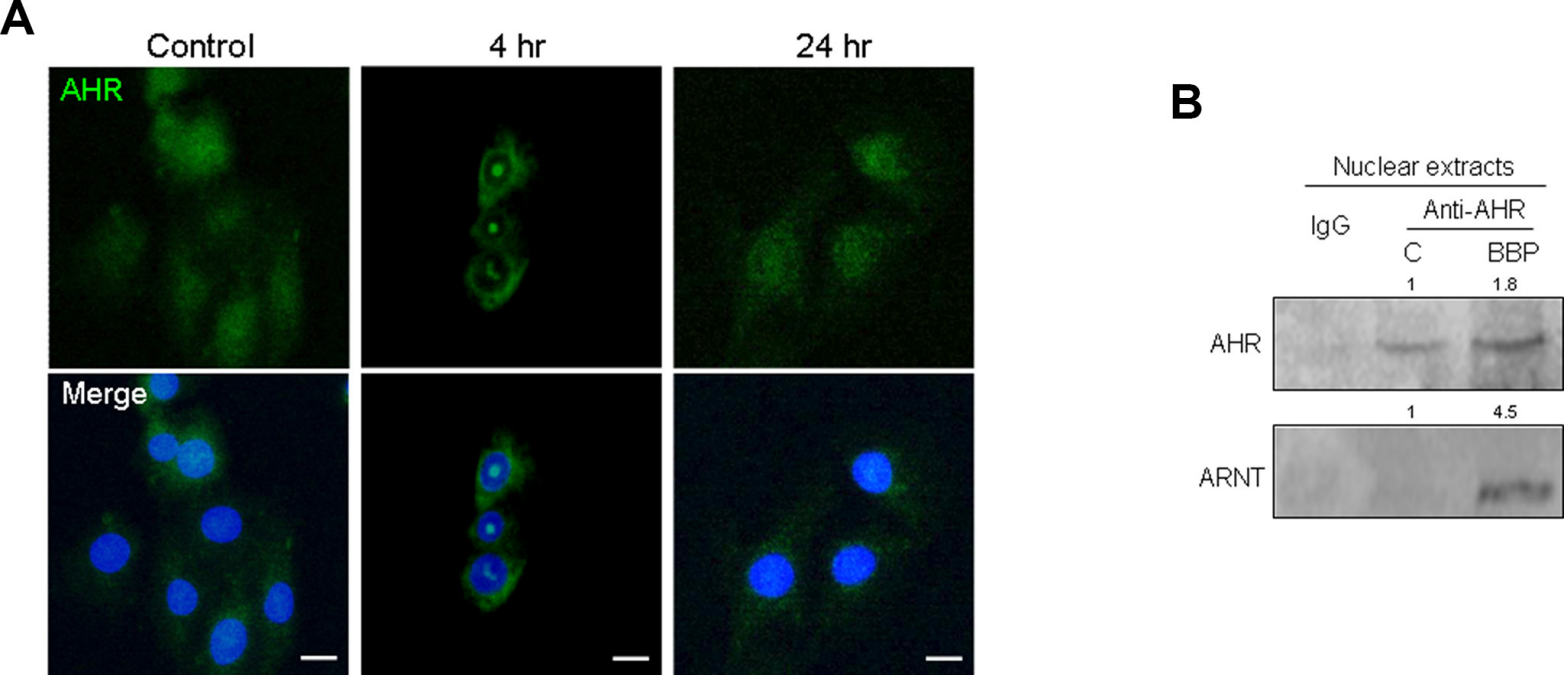
BBP-stimulated AHR nuclear accumulation and ARNT-binding MCF-7 cells were treated for 24 h with 1 μM BBP. Cells were fixed and AHR distribution was detected by indirect IF microscopy. (**A**) Nuclei (blue) are labeled with DAPI. Scale bars = 20 μm. AHR/ARNT complex detection in BBP-treated MCF-7 cell nuclear extracts. (**B**) Band intensity was quantified by densitometry and values are expressed relative to the control group.

**Figure 3:**
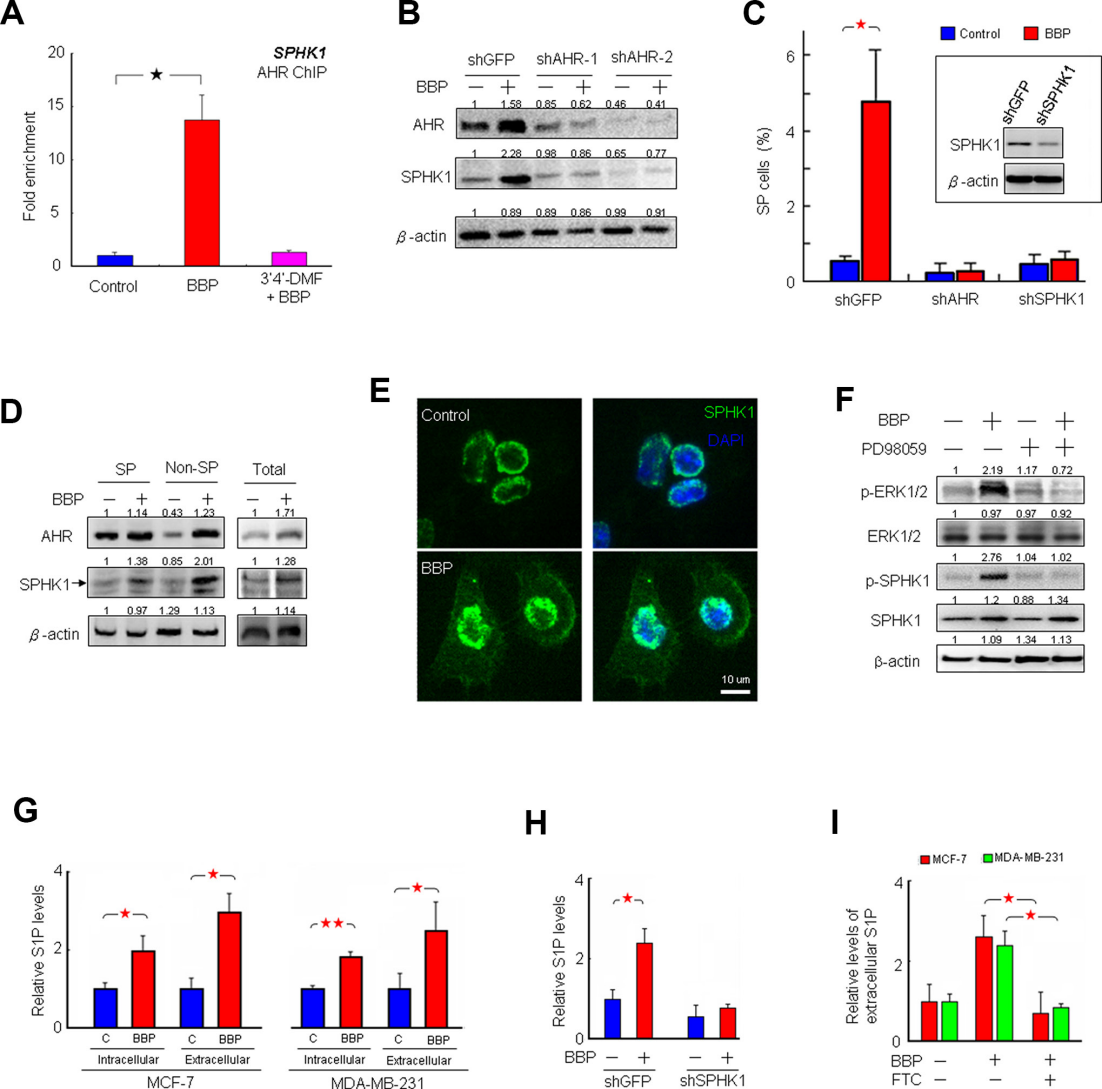
BBP induces SPHK1 expression and activity and triggers S1P release BBP-induced AHR targeted *SPHK1* gene transcription in MCF-7 cells as shown by ChIP-qPCR assay, and this was blocked by AHR inhibitor 3ʹ4ʹ-DMF (*n* = 4). (**A**) Representative AHR and SPHK1 immunoblots with lysates of MCF-7 cells transfected with control or AHR shRNA, with or without BBP. (**B**) β-actin was used as a loading control. Band intensity was quantified by densitometry and values are expressed relative to the control group. SP assays of MCF-7 cells transfected with control, AHR or SPHK1 shRNA, with or without BBP. (**C**) Inset box shows SPHK1 levels in control and SPHK1 shRNA-transfected MCF-7 cells by western blot. Western blot analysis of AHR and SPHK1 (arrow) signaling in SP and non-SP cells separated from the MCF-7 cell lines. (**D**) MCF-7 cells with or without BBP were stained for DAPI (nuclei blue) and SPHK1-Alexa Flour 488 (green) and examined by confocal fluorescence microscopy. (**E**) Western blot analysis of ERK (ERK1/2), phospho-ERK (p-ERK1/2), SPHK1 and phospho-SPHK1 (p-SPHK1) in MCF-7 cells treated with PD98059 (50 μM) and BBP (**F**) β-actin was used as a loading control. S1P levels in both the intracellular extract and extracellular medium of MCF-7 and MDA-MB-231 cells after overnight BBP treatment measured via ELISA (*n* = 5). (**G**) S1P levels in the intracellular extract of MCF-7 cells transfected with control or SPHK1 shRNA, with or without BBP. (**H**) S1P levels in the extracellular medium of MCF-7 and MDA-MB-231 cells treated with BBP plus FTC (**I**) Data are presented as mean ± SD; *P < 0.05; **P < 0.01.

We previously showed that ligand-dependent AHR activation is indicative of proliferation and invasiveness in MDA-MB-231 cells [2]. IF staining showed AHR nuclear translocation in MCF-7 cells treated with BBP (Figure 2A). A nucleocytoplasmic fractionation assay indicated that BBP-activated AHR formed a complex with ARNT in MCF-7 cell nuclei (Figure 2B). These results suggested that BBP could be a potent AHR ligand. A previous study showed that SPHK1/S1P is associated with BCSC expansion [5]. Our finding that BBP increased SPHK1 expression in a dose-dependent manner (Figure S1A) prompted us to examine correlations between SPHK1 and AHR. To identify potential XREs, the region between the −2000 and +500 positions of SPHK1 promoter was analyzed using the LASAGNA-Search 2.0 transcription factor regulatory element prediction software [17], which yielded two potential transcription factor-binding domains in the region (Figure S1B). Chromatin immunoprecipitation (ChIP) assays showed that AHR bound to the promoter of *SPHK1* (Figure 3A). AHR-induced SPHK1 synthesis was confirmed using the AHR inhibitor, 3ʹ,4ʹ-dimethoxyflavone (3ʹ4ʹ-DMF), (Figures 3A, S1C–S1D) and AHR short hairpin RNAs (shRNAs) (Figure 3B). These results suggested that AHR transcriptionally activated SPHK1. Additionally, shAHR and shSPHK1 inhibited BBP-induced SP cell expansion (Figure 3C). These results indicated that AHR/SPHK1 signaling was required for SP cell expansion.

Western blotting results showed that AHR and SPHK1 expression was higher after BBP treatment in both SP and non-SP MCF-7 cells (Figure 3D). IF analysis showed that BBP stimulation increased both membrane-associated SPHK1 levels and activation by phosphorylation at serine 225 (Figure 3E–3F). A previous report showed that SPHK1 membrane translocation activated by extracellular signal-regulated kinase (ERK) 1 and 2 both increases intracellular S1P and enhances the release of S1P into the extracellular environment [18]. In the current study, BBP treatment was associated with increased ERK phosphorylation (Figure 3F). The ERK inhibitor, PD98059, blocked BBP-induced increases in ERK1/2 and SPHK1 phosphorylation. Enzyme-linked immunosorbent assay (ELISA) showed that activated SPHK1 increased intracellular S1P and enhanced S1P release into the medium for both MCF-7 and MDA-MB-231 cells (Figure 3G). BBP-induced S1P production and release were prevented by SPHK1 knockdown (Figure 3H) and the ABC transporter inhibitor fumitremorgin C (FTC) (Figure 3I), respectively.

### SPHK1 promotes breast cancer cell metastasis to the lungs

**Figure 4:**
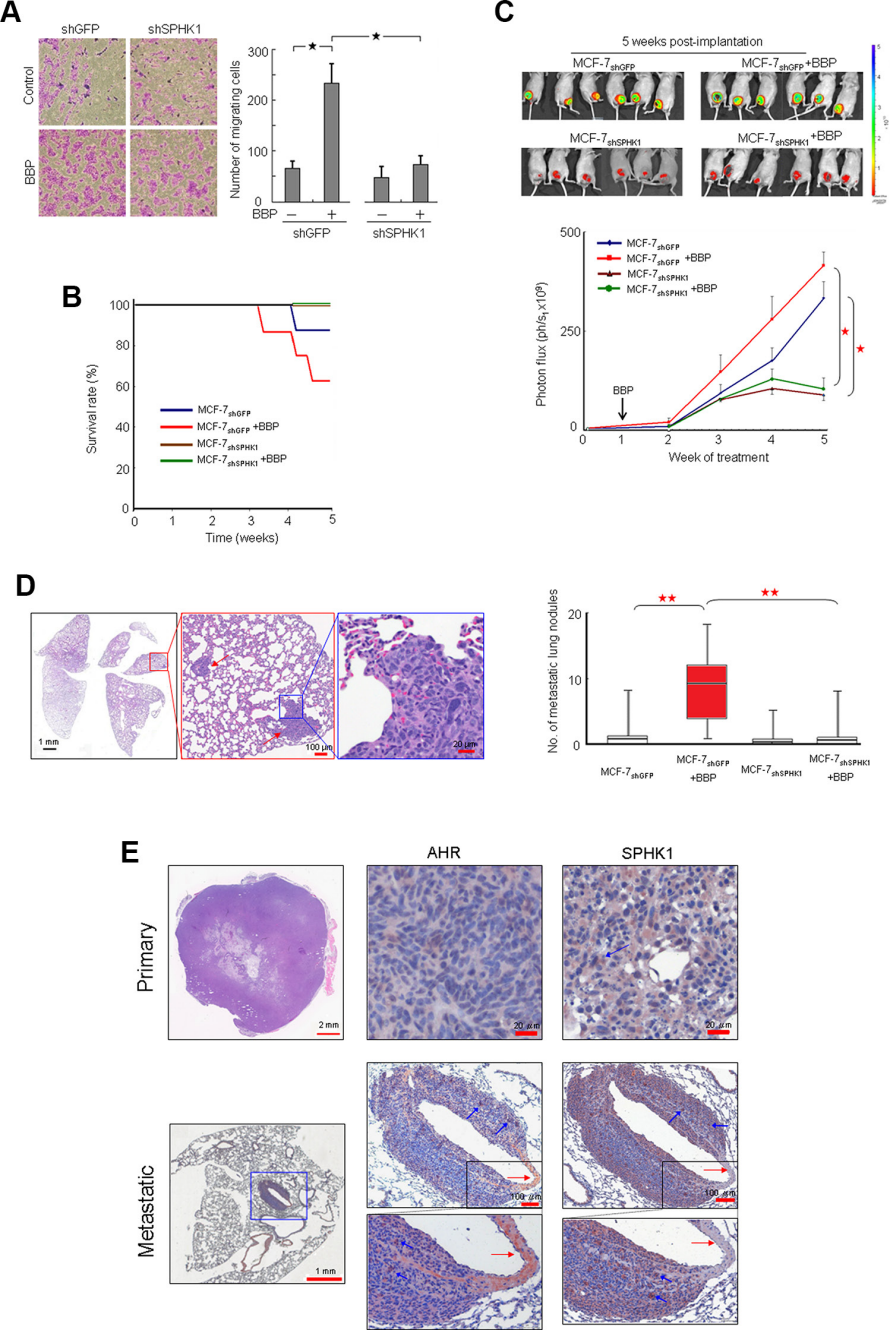
Promotion of SPHK1 during breast tumorigenesis and metastasis Phase-contrast images from the transwell migration assay after SPHK1 knockdown (left panel) (**A**) The number of migrating cells was computed and graphed (right panel). MCF-7_shSPHK1_ or MCF-7_shGFP_ cells were implanted in ovariectomized Nu/Nu immunodeficient mice, and BBP (200 mg/Kg body weight) or corn oil were administered by intraperitoneal injection. Survival rate (**B**), tumor size (**C**), and number of metastatic lung nodules (**D**) were compared with those of control and BBP-treated mice (*n* = 6 in each group) at 5 weeks post-implantation (upper panel). The luminescent signals (photon flux) of transplanted cells were plotted over time and revealed tumor growth inhibition in MCF-7shSPHK1 vs. MCF-7shGFP lines (lower panel) (*P* < 0.05). HE staining of a lung metastatic nodule (red arrow) is shown at three magnifications (left panel). The red box indicates the sample distribution between 25% and 75% (*n* = 8). HE staining of primary and metastatic tumors together with AHR and SPHK1 IHC staining shows AHR and SPHK1 co-expression in metastatic breast cancer cells (blue arrows). Data are presented as mean ± SD; *P < 0.05; **P < 0.01.

BBP induced cell migration in MCF-7 cells, and SPHK1 knockdown inhibited these effects (Figure 4A). To investigate whether SPHK1 can promote metastasis through EMT, MCF-7 cells were transfected with recombinant lentivirus vectors carrying shRNA specific for SPHK1 (MCF-7_shSPHK1_) or control shRNA (MCF-7_shGFP_). After viral transduction and puromycin selection, transduced cells were transplanted into the mammary fat pads of nude mice. SPHK1 expression in MCF-7_shSPHK1_ cells was less than that in MCF-7_shGFP_ cells (Figure S2A). Mice that received MCF-7_shSPHK1_ cells exhibited disease-free survival 5 weeks post implantation compared with mice that received MCF-7_shGFP_cells (Figure 4B). Tumor growth was monitored using caliper measurements and IVIS quantitative imaging of tumors expressing EGFP (Figure 4C). MCF-7_shSPHK1_ cells exhibited reduced growth compared with MCF-7_shGFP_ cells *in vivo* (Figure 4C). Histological analysis showed that BBP-treated MCF-7_shGFP_ mice had increased metastatic lung lesions compared with untreated MCF-7_shGFP_ mice, but BBP treatment did not correlate with the number of metastatic lung nodules in MCF-7_shSPHK1_ cell-implanted mice Figure 4D). Immunohistochemistry (IHC) revealed that the number of stained AHR and SPHK1 cells was reduced in the primary tumor. However, there were more double-positive cells in the metastatic tumor mass around the invading blood vessel (Figure 4E) in what appeared to be epithelial-like cells embedded in the tumor (Figure S2B).

### S1PR3 signaling expands the BCSC population

**Figure 5:**
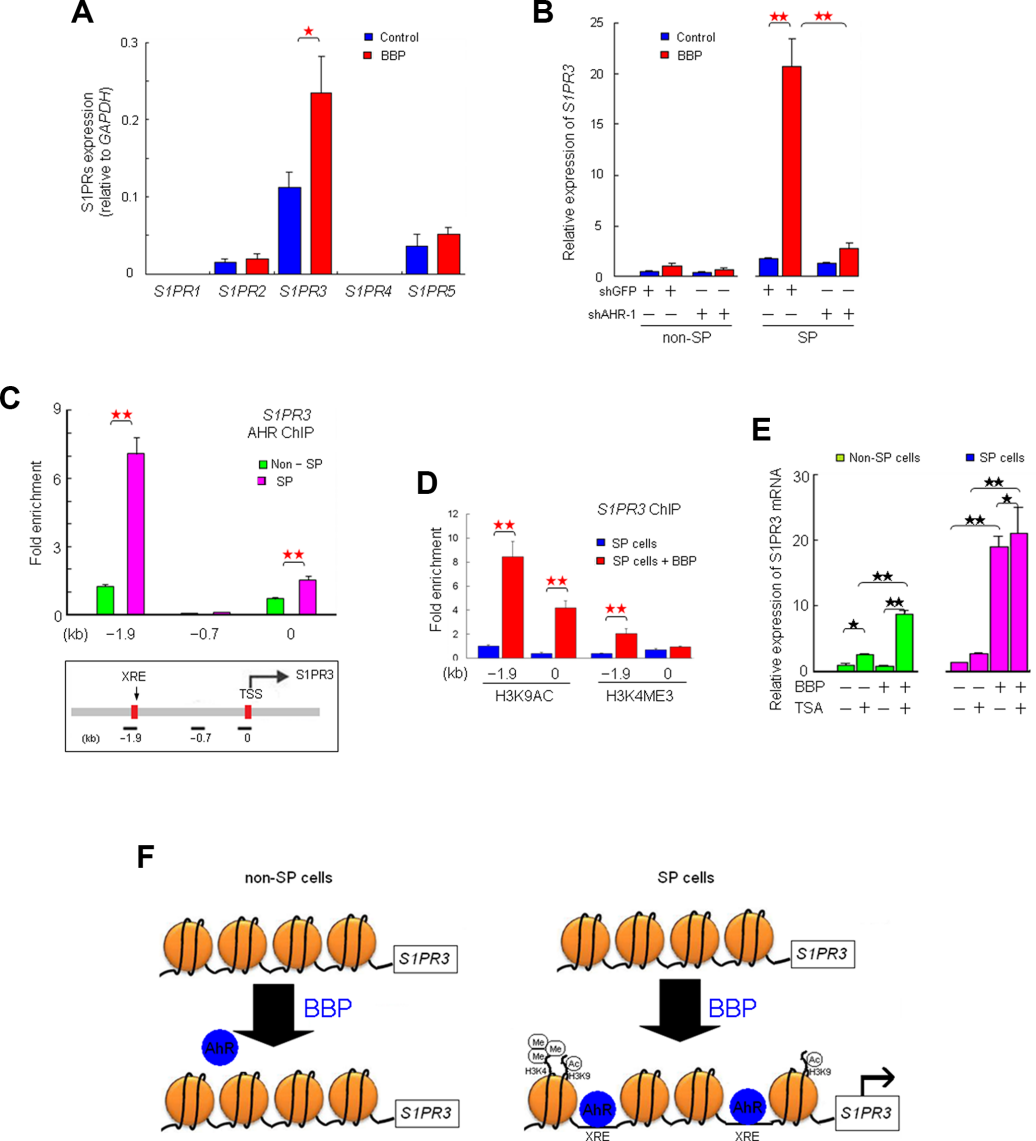
AHR activates *S1PR3* in SP, but not non-SP cells *S1PR1*–*5* mRNA levels in non-SP and SP MCF-7 cells after BBP stimulation, as determined by qRT-PCR (**A**) BBP-induced *S1PR3* mRNA elevation in SP, but not non-SP, MCF-7 cells. (**B**) Relative fold enrichment of AHR (over IgG control) bound at two sites of the *S1PR3* promoter in SP cells as determined by ChIP assay. (**C**) Values were normalized to the total input DNA. Lower panel: linear scale map of the *S1PR3* locus with the location of XREs (red boxes), transcription start site (TSS) and PCR amplicons used in the ChIP assay (short black bars). Relative fold enrichment of H3K4ME3 and H3K9AC (over IgG control) on promoter regions in SP cells stimulated with BBP for 1 day. (**D**) *S1PR3* expression in TSA-treated SP or non-SP cells, with or without BPP (**E**) Simplified model of BBP-induced epigenetic regulation of *S1PR3* expression. (**F**) In SP cells, BBP-induced H3K4ME3 and H3K9AC at the *S1PR3* promoter regions may promote XRE-AHR binding, which activates *S1PR3* expression. Data are presented as mean ± SD from four replicates; *P < 0.05; **P < 0.01.

Extracellular S1P binds S1PR at the cell membrane. Quantitative real-time PCR (qRT-PCR) revealed that *S1PR3* was the most abundant S1PR in MCF-7 cells; small quantities of *S1PR2* and *S1PR5* were present, and *S1PR1* and *S1PR4* were not detected (Figure 5A). *S1PR3* expression was elevated in BBP-treated SP cells, but not in non-SP cells (Figure 5B). AHR knockdown blocked BBP-induced S1PR3 increases in SP cells (Figure 5B). ChIP showed that two *S1PR3* promoter fragments containing putative AHR binding sites were enriched in BBP-treated SP cells, but not in non-SP cells (Figure 5C).

Hung, *et al.* [19] showed that phthalate exposure induces histone modification, such as WDR5 (lysine 4 of H3 histone-specific trimethyltransferase), and modulates gene expression epigenetically. To investigate whether epigenetic control was involved in *S1PR3* transcriptional regulation, the relative abundance of active modification, such as lysine 9 acetylation of H3 histone (H3K9AC) and lysine 4 trimethylation of H3 histone (H3K4ME3) [20], was measured on the *S1PR3* promoter by ChIP. BBP induced H3K9AC and H3K4ME3 at the XRE sites (regions −5 to +77 and −1907 to −1732) of the *S1PR3* promoter region in SP cells (Figure 5D), but not in non-SP cells (data not shown). Trichostatin A (TSA, histone deacetylase inhibitor) alone or in combination with BBP induced *S1PR3* mRNA elevation in non-SP cells (Figure 5E). These results suggested that BBP induced epigenetic regulation via histone modification in SP cells to promote AHR transcriptional activation of *S1PR3*. *S1PR3* promoter histone modifications in SP and non-SP cells are shown in Figure 5F.

**Figure 6:**
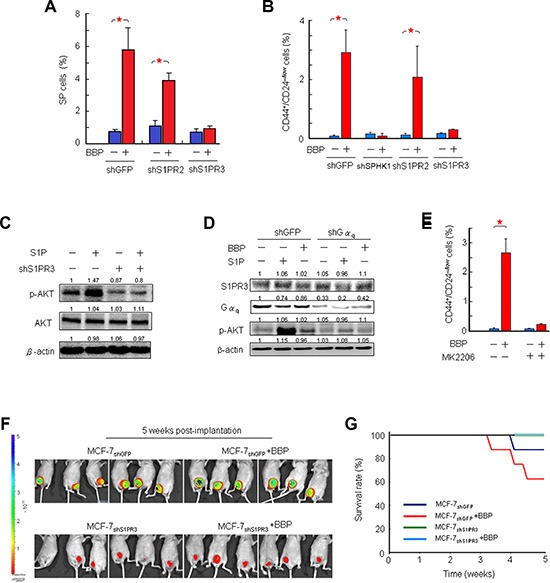
Correlation between S1PR3 and BCSC-mediated tumor formation in a mouse xenograft model Effects of S1PR2 and S1PR3 shRNAs on BBP-induced MCF-7 SP cell proliferation (**A**) Effects of SPHK1, S1PR2 and S1PR3 shRNAs on BBP-induced CD44^+^/CD24^−/low^ MCF-7 cell proliferation. (**B**) Effects of S1PR3 shRNAs on S1P-induced AKT activation. (**C**) Western blotting for total AKT and phospho-AKT (p-AKT) was performed with cell lysates obtained after S1P (100 nM, 1 h) treatment. β-actin was used as a loading control. Effects of Gα_q_ shRNAs on S1P- or BBP-induced AKT activation (**D**) MCF-7 cells were treated with S1P (100 nM) or BBP (1 μM) after transfection with control or Gα_q_ shRNA. β-actin was used as a loading control. Effects of MK2206 (AKT inhibitor, 50 nM) on BBP-induced CD44^+^/CD24^−/low^ MCF-7 cell proliferation (**E**) MCF-7_shS1PR3_ or MCF-7_shGFP_ cells were implanted in ovariectomized Nu/Nu immunodeficient mice, and BBP (200 mg/Kg body weight) or corn oil were administered by intraperitoneal injection. Tumor size (**F**) and survival rate (**G**) were compared with those of control and BBP-treated mice at 5 weeks post-implantation. Representative bioluminescent images of tumor growth at 5 weeks post-implantation are shown (upper panel). Data are presented as mean ± SD from three replicates; *P < 0.05.

We next investigated whether S1PR3 signaling was necessary for BBP-induced BCSC expansion. Knockdown of S1PR3, but not S1PR2, abolished the BBP-expanded SP and CD44^high^/CD24^low^ MCF-7 cell populations (Figures 6A–6B and S3). S1P-triggeed responses are often mediated by coupling of G-protein-coupled receptors to different heterotrimeric G proteins. S1PR3 couples with G12/13, Gα_q_ and Gα_i_, and these G proteins activate downstream signals such as AKT phosphorylation [21]. Consequently, we tested the ability of S1P to activate AKT in MCF-7 cells. S1P increased AKT serine 473 phosphorylation and S1PR3 knockdown inhibited this effect (Figure 6C). BBP- and S1P-induced AKT activation was blocked by shRNA targeted to Gα_q_ (Figure 6D). These results indicated that S1PR3 may be a major receptor in the signaling pathway that results in AKT phosphorylation induced by S1P through Gα_q_. BBP-induced expansion of the CD44^high^ CD24^low^ cell population was blocked by the AKT inhibitor MK2206 (Figure 6E). This was supported by an *in vivo* experiment showing that implantation of MCF-7_shS1PR3_ cells into the mammary fat pads of mice could eliminate BBP-induced breast tumors at 5 weeks post-implantation (Figures 6F and S4). These mice had a prolonged survival rate (Figure 6G), suggesting that S1PR3 could be a potential prognostic biomarker in breast cancer.

## DISCUSSION

In the present study, we determined that AHR/SPHK1/S1P signaling and subsequent S1PR3 activation increased BCSCs. Knockdown of SPHK1 or S1PR3 reduced breast cancer cell tumorigenicity. BBP-activated AHR targeted *S1PR3* transcription in SP, but not in non-SP cells. Thus, S1P/S1PR3 signaling initiated in SP cells could produce metastasis-initiating BCSCs. BCSCs are relatively resistant to chemotherapeutics [22] and are responsible for cancer metastasis [23, 24], which leads to death in the majority of breast cancer patients. Collectively, these data will determine the extent to which BBP influences BCSC signaling pathways and help to elucidate the impact of chemical exposure on cancer malignancy.

AHR expression can be detected as early as gestational day 10 in mouse embryos [25]. AHR-deficient mice exhibit a 50% decrease in mammary gland terminal end buds [26]. Evidence suggests that these alterations in mammary development are permanent in developing embryos exposed to AHR agonists *in utero* [27]. AHR overexpressing mice are prone to stomach tumors and intestinal metaplasia precancerous lesions [28]. AHR knockout mice exhibit decreased tumorigenicity and cell migration due to Vav3 and Rac1 activity downregulation [29]. Shimizu, *et al.* [30] found that benzo(a)pyrene carcinogenicity was reduced in AHR-deficient mice. AHR signaling promotes the BCSC population in tamoxifen-resistant MCF7 cells but does not affect BCSCs in tamoxifen-sensitive cells [9].

Whether or not different AHR ligands induce different or even opposite biological outcomes remains controversial [31, 32]. BBP may specifically activate the oncogenic AHR gene cluster while inhibiting or not influencing the tumor-suppressive AHR gene cluster. The opposing roles for AHR in tumorigenesis documented in the literature may be reconciled by the hypothesis that different AHR ligands may activate or suppress distinct gene clusters, of which some are oncogenic and some are tumor-suppressive. Further studies are needed to test this hypothesis.

JARID1B (H3K4 demethylase) correlates with metastatic progression based on xenotransplant assays in immunodeficient mice [33]. Polychlorinated biphenyl activates JARID1B through AHR and androgen receptor signaling [34]. The H3K9AC modification is mediated by acetyltransferases, such as PCAF or GCN5 [35]. We found that H3K9AC and H3K4ME3 specifically accumulated on the *S1PR3* promoter, indicating that S1PR3 overexpression in SP cells is epigenetically linked. This suggests that phthalates induce direct epigenetic alterations in exposed cells [36–38], but does not explain why histone modification did not occur in non-SP cells. Previous reports showed that histone modification enzymes, such as enhancer of Zeste Homolog 2 (EZH2), are expressed at higher levels in BCSCs than non-BCSCs [39]. Further study is needed to investigate whether levels of endogenous histone modification enzymes impact BBP effects in BCSCs and non-BCSCs.

AHR acts as a modulator in both SP and non-SP cells during primary tumor growth. BPP-induced histone modifications correlated with S1PR3 expression in SP and non-SP cells. AHR activation induced S1PR3 overexpression in SP cells, but only a mild increase in non-SP cells. Gradual S1PR3 accumulation may promote BCSC proliferation and invasion compared to non-BCSCs. This report represents the first functional characterization of AHR as a phthalate-induced SPHK1 activator, leading to S1P production. Computational analysis of the *SPHK1* promoter revealed putative AHR binding sites (S1C). Combined with ChIP assays, shRNA and AHR inhibitor results identified a novel pathway whereby AHR activates SPHK1 in response to BBP stimulation. SPHK1 [40] and S1PR3 [14] expression in human breast cancer tissue is associated with reduced time to recurrence and cumulative disease-specific survival. S1PR3 acts as a chemotactic receptor and mediates S1P activation of AKT [41, 42]. AKT correlates with cancer stem cell survival and tumorigenesis [43]. S1PR3 or Gα_q_ knockdown significantly abolishes S1P-induced AKT activation [41], indicating that S1P-activated S1PR3 and Gα_q_ mediate AKT activation in MCF10A cells. S1P/S1PR3 triggered Gα_q_–AKT activation is required for BBP-induced BCSC expansion (Figure 6C–6D). Our results implicate S1PR3 as a potentially valuable therapeutic target for regulating BCSCs in breast cancer patients. Furthermore the association between breast carcinogenesis and environmental pollutants has important implications for public health.

## MATERIALS AND METHODS

### Cell culture

Human breast cancer cell lines MCF-7 and MDA-MB-231 were cultured in DMEM and DMEM/F12 medium with 10% fetal bovine serum (FBS), respectively (Invitrogen, Carlsbad, CA) and incubated in a humidified atmosphere with 5% CO_2_ at 37°C.

### Transwell migration assay

An *in vitro* migration assay was performed using a 24-well Transwell unit with polycarbonate filters (8-μm pore size; BD Biosciences, Franklin Lakes, NJ, USA), as previously described with slight modifications [44]. The upper chamber contained cells in culture medium (1 × 10^4^/ml) with 1% FBS, and the lower chamber contained culture medium with 10% FBS. After 1 day, the medium and non-migrated cells were removed from the top chamber using cotton swabs and phosphate buffered saline (PBS). Migrated cells remaining on the bottom surface were counted after staining with 0.5% crystal violet solution for two h.

### Small hairpin RNA (shRNA) transfection

shRNA targeting AHR (TRCN0000245283, TRC N0000245285 and TRCN0000021255), SPHK1 (TRCN 0000036965, TRCN0000036968 and TRCN0000219837), S1PR2 (TRCN0000011382, TRCN0000221136 and TRCN 0000221138), S1PR3 (TRCN0000221126, TRCN0000 221128 and TRCN0000356946) and control shRNA (shGFP; TRCN0000072178) were purchased from National RNAi Core Facility (NRCF), Taiwan. Cells were plated in 6-well plates at 1 × 10^5^ cells/well. Transfection was performed using TransIT-LT1 Transfection Reagent (Mirrus Bio, Madison, WI, USA) and OPTI-MEM I (Invitrogen, Carlsbad, CA). Cells were transfected with siRNA or shRNA at 2 μg plus 4 μl transfection reagent. Control cells were treated with negative control shRNA (shGFP).

### Stable transfection

shRNA cloning lentivector (pLKO-TRC011), package plasmids (pMD.G, pCMVDR8.91 and pLAS2w.RFP-C.Ppuro) was were purchased from NRCF. To generate lentiviral shRNA constructs (pLKO-shSPHK1 and pLKO-shS1PR3), the shSPHK1 or shS1PR3 sequences were cloned into the pLKO-TRC011 vector. Inserted sequences were confirmed by DNA sequencing. To generate the SPHK1- and S1PR3-knockdown cell lines (MCF-7_shSPHK1_ and MCF-7_shS1PR3_), lentiviral particles were produced by co-transfection of 293T cells with lentivector (pLKO-TRC011, pLKO-shSPHK1 or pLKO-shS1PR3) and lentiviral packaging vector mixed according to standard protocols (NRCF). Lentivirus-containing supernatant was harvested 48 h post-transfection, purified by centrifugation, and stored at −80°C. For viral transduction, the lentivirus was incubated with MCF-7 cells in the presence of 4 μg/ml polybrene overnight at 37°C. Cells were screened for target expression using puromycin (2 μg/ml) for 7 days. After 2 weeks of stable growth in complete medium, target knockdown was verified by western blotting.

### Side population assay, flow cytometry and FACS

BBP-treated or control cells (1 × 10^6^ /ml) were suspended in pre-warmed DMEM medium and incubated with Hoechst 33342 (Invitrogen H-3570, 5 μg/ml) for 90 min at 37°C. Cells treated with verapamil (Sigma, 50 μM) were used as a negative control. After incubation, propidium iodide (2 μg/ml) was added (5 min) to discriminate dead cells from live cells. Samples were sorted using a MoFlo XDP cell sorter (Beckman Coulter, Fullerton, CA). The Hoechst dye was excited with a UV laser at 351 to 364 nm, and fluorescence was measured with 425-nm (Hoechst blue) and 650-nm (Hoechst red) filters. In FACS, the gate for the SP was set for cells that appeared as a tiny population on the lower right-hand side of the Hoechst blue (*x* axis) against Hoechst red (*y* axis) plot. The SP gate of the flow analysis was established and defined using control cells stained with both Hoechst and verapamil. Non-SP cells were gated at the center of the main population.

To determine the CD44^high^CD24^low^ phenotype, cells were trypsinized, washed, and resuspended at 1 × 10^6^ cells/ml in 0.5% bovine serum albumin (BSA) in PBS containing diluted anti-human CD44 (phycoerythrin (PE) conjugated) and anti-human CD24 (fluorescein isothiocyanate (FITC) conjugated) (BD Biosciences). Cells were analyzed using a LSR II flow cytometer (Becton Dickinson, San Jose, CA) and CellQuest (BD Biosciences) or Summit 5.3 (Becton Dickinson) software.

### Nucleocytoplasmic subcellular fractionation and co-immunoprecipitation

Nuclear and cytoplasmic fractions of MCF-7 cells treated with DMSO or BBP for 24 h were collected by nuclear extract kit (Active Motif, Rixensart, Belgium). Immunoprecipitations (IP) were performed by adding anti-AHR monoclonal antibody or IgG to 200 μg of cell lysate. Lysates were incubated with antibodies overnight at 4°C before addition of 30 μl Protein A beads (Amersham Pharmacia Biotech), followed by a second overnight incubation at 4°C. Beads were washed with IP wash buffer and bound proteins were released by boiling in 1X gel loading buffer. Fractionation efficiency was tested by western blotting for α-tubulin and Histone H3.

For western blotting, proteins were separated by 8% SDS-polyacrylamide gel electrophoresis and transferred to polyvinylidene difluoride (PVDF) membranes (Invitrogen). After blocking with 5% non-fat dry milk in PBS, the membrane was washed and incubated overnight at 4°C with primary antibody. The appropriate HRP-conjugated second antibody was added after PBS-tween20 washing. Bands were visualized using Immobilon Western Chemiluminescent HRP Substrate (Millipore). Proteins were stripped from the blotting membrane by incubation in Restore PLUS Western Blot stripping buffer (Thermo Scientific).

### Immunofluorescence and immunohistochemical staining

For IF staining, sorted cells were seeded on glass coverslips in a 24-well plate, fixed with 2% formaldehyde for 15 min and permeabilized in methanol for 10 min at −20°C. Primary antibody was added and incubated overnight at 4°C followed by 5% BSA in PBS for 1 h. After washing in PBS, Alexa 488– or Alexa 594–conjugated secondary antibody was added in the dark for 60 min at room temperature (RT). Finally, cells were incubated with 4ʹ,6ʹ-diamidino-2-phenylindole (DAPI, 1 μg/ml) for 10 min at RT. Imaging was conducted using a TissueFaxs system (TissueGnostics, Austria) and FV-1000 confocal microscope (Olympus, Japan).

For IHC staining, tissues were fixed in 4% paraformaldehyde, embedded in paraffin and cut into 4-μm-thick sections. Sections were rehydrated and antigens were retrieved in sodium citrate buffer (10 mM, pH 6.0). After blocking with Peroxidase Blocking Reagent (Dako) and 5% normal goat or rabbit serum (Invitrogen) in PBS, sections were incubated overnight at 4°C with primary antibodies. Slides were washed and incubated with horseradish peroxidase–conjugated anti-rabbit or anti-mouse antibodies (Dako) for 1 h at RT The signal was developed using 3, 3-diaminobenzidine and slides were counterstained with hematoxylin. Images were taken using the TissueFaxs system.

### ChIP

Specific antibodies were used to immunoprecipitate DNA-containing complexes from formaldehyde cross-linked MCF-7 cells (1 × 10^7^ cells). SP and non-SP cells sorted by FACS for ChIP analysis (Upstate Biotechnology) were seeded onto 6-well plates (1 × 10^4^ cells/well) and cultured to 70% confluence in DMEM with 10% FBS. PCR was conducted with primers complementary to the SPHK1 or S1PR3 promoter regions. Amplified products were loaded onto 2% agarose gels and stained by ethidium bromide. For the human *SPHK1* and *S1PR3* promoter regions, primer sets (for *SPHK1*: 5ʹ-GGAGGAAGAAAGAGGGAAGC-3ʹ and 5ʹ-ACCCTTGGTTTCACCTCGAC-3ʹ; 5ʹ-GGTCCT CCGGAAGAGAAGAC-3ʹ and 5ʹ-CAGGTAGGGCCAG AGTTAGG-3ʹ; for *S1PR3*: 5ʹ-TTTAGGCAAACGGAG CCTCA-3ʹ and 5ʹ-CAACCTTGAGGCGTGGTGAT-3ʹ; 5ʹ-TGTTCGCTCAATCATGGCCT-3ʹ and 5ʹ-ATTTGCCC CTTTTGTGTGGC-3ʹ; 5ʹ-TTTAGTCTCTGACTCGCT CGGG-3ʹ and 5ʹ-GCCTTCTGGTCCCTGAGTCC-3ʹ; 5ʹ-GTGTGCTATGTCCATGGTGC-3ʹ and 5ʹ-AGGCTACC GGAGATCCTTCC-3) were used for qRT-PCR. Genomic DNA sampled before adding the antibody (input) was used as a positive control; IgG immunoprecipitate was used as a negative control. The amount of immunoprecipitated DNA in each sample is represented as signal relative to the total amount of input chromatin.

### qRT-PCR

To assess expression of S1PRs, total RNA from sorted cells was extracted using an RNeasy mini kit (Qiagen, Valencia, CA, USA). Briefly, RNA (1 μg) was quantified with a NanoDrop spectrophotometer (Wilmington, DE, USA), treated with DNase and reverse transcribed to cDNA using a first-strand cDNA synthesis kit (Promega, Madison, WI). Reactions were carried out using TaqMan^®^ Gene Expression Master Mix (Applied Biosystems, Foster City, CA, USA) on an ABI 7500 system (Applied Biosystems). Each reaction was performed in triplicate. All primer sets amplified fragments < 200 bp long. Primers are shown in Table S3. The amount of each target gene in a given sample was normalized to the level of 18S rRNA or GAPDH mRNA in that sample.

### ELISA

An S1P competitive ELISA kit (Echelon Biosciences Inc., Salt Lake City, UT, USA) was used to measure S1P levels according to manufacturer's instructions.

### Animal experiments

The animal study was approved by the Institutional Animal Care and Use Committee, Kaohsiung Medical University (Approval Number: IACUC-101059). All animals were purchased from the National Laboratory Animal Center (Taipei, Taiwan) and maintained in standard conditions according to institutional guidelines. After sorting, SP and non-SP MCF-7 cells were counted and re-suspended in culture medium. Subcutaneous injections were performed in female nude athymic (nu/nu) mice (6–8 weeks old) on the dorsal flank under isoflurane anesthesia. Tumor development was monitored weekly. When tumor burden became obvious, the experiment was terminated.

MCF-7_shGFP_, MCF-7_shSPHK1_ or MCF-7_shS1PR3_ cells (1 × 10^6^ in 40 μl of medium:Matrigel (1:1)) were injected into mammary fat pads of female nude mice (6–8 weeks old; *n* = 6 in each group) under isoflurane anesthesia. To adapt to the estrogen depletion changes, mice were ovariectomized 1 week before tumor implantation. Estrogen capsules (silastic tube filled with 1 mg/ml estradiol in corn oil) were placed under the skin of each mouse after ovariectomization. Intraperitoneal injection of BBP (200 mg/Kg body weight, 3× per week) was initiated at one week after cell implantation. Tumor growth was monitored weekly using a bioluminescence imaging system under isoflurane anesthesia. Data were collected as photons/sec/cm^2^ using Living Image software (PerkinElmer Inc., Waltham, MA, USA). Mice were sacrificed 5 weeks after tumor implantation. Primary tumors together with several other tissues/organs (i.e. lymph nodes, lung, intestine, stomach, liver, spleen, and kidney) were removed, weighed, washed in PBS, formalin fixed in 10% formaldehyde and processed for paraffin embedding for hematoxylin and eosin (HE) staining and IHC analysis. The number of lung metastases was counted under a dissecting microscope.

### Statistic analysis

Datas are expressed as the mean ± SD. Results were analyzed by Student's *t*-test using SPSS 13.0 software package (SPSS, Inc., Chicago, IL, USA). A two-tailed *P* < 0.05 was considered as statistically significant.

## SUPPLEMENTARY MATERIALS FIGURES


