# Supplementary material for: Benzyl butyl phthalate promotes breast cancer stem cell expansion via SPHK1/S1P/S1PR3 signaling
Source: Oncotarget. 2016 Apr 26;7(20):29563–76. doi: 10.18632/oncotarget.9007 (PMC5045417; doi:10.18632/oncotarget.9007)
Supplement: Supplementary file 1 [file oncotarget-07-29563-s001.pdf]

# Benzyl butyl phthalate promotes breast cancer stem cell expansion via SPHK1/S1P/S1PR3 signaling

## Supplementary Materials

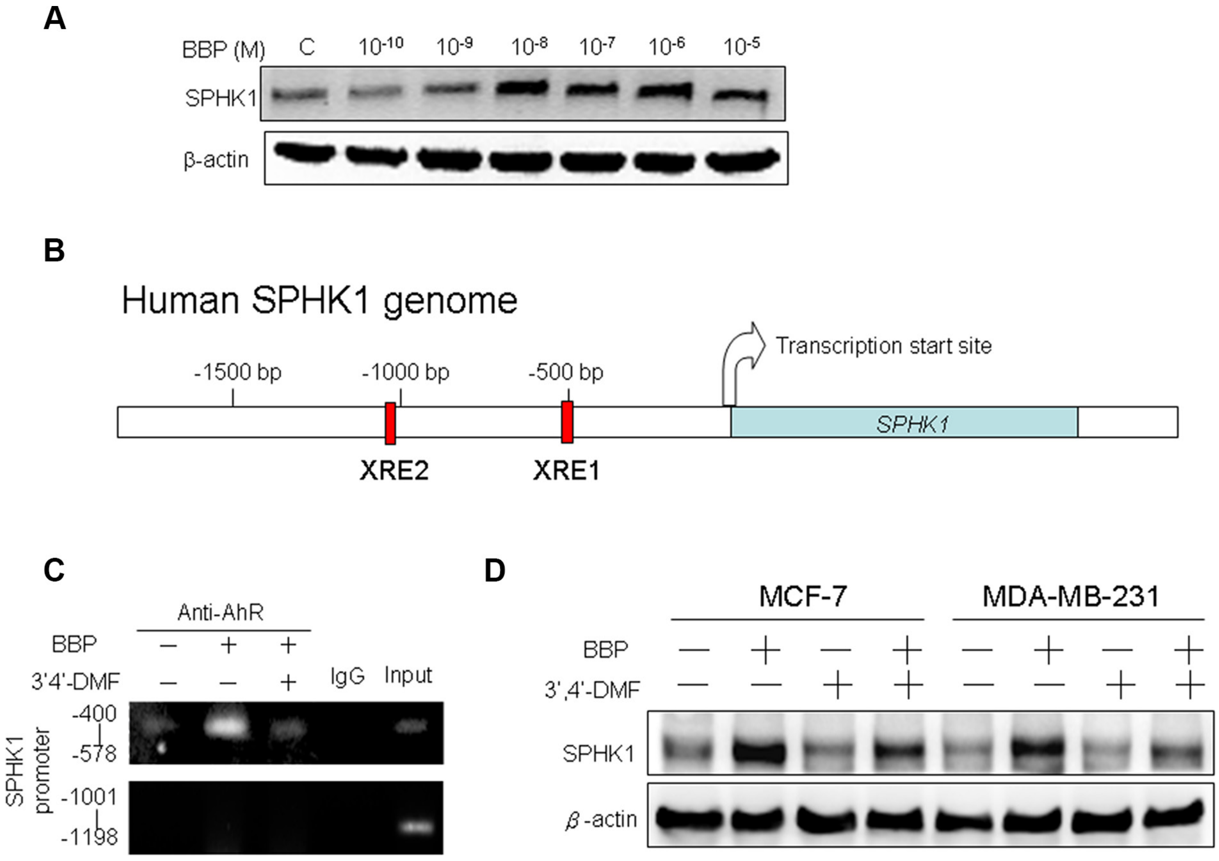


**Supplementary Figure S1:** (**A**) Western blot showing SPHK1 expression in MCF-7 cells after 24 hours treatment with the indicated concentrations of BBP. (**B**) Sequence alignment of human *SPHK1* regulatory regions containing 2 putative XREs and locations of PCR amplicons used in the ChIP assay (red boxes). (**C**) ChIP analysis revealed that BBPinduced AhR target at the promoterof *SPHK1*. Pretreated with 3′4′DMF reduced the BBP-induced AhR binding to the *SPHK1* promoter. IgG as negative control, Input indicating positive control. (**D**) Western blot analysis showed that BBP induced SK1 expression in both MCF7 and MDAMB231 cells. These increases were inhibited by 3′4′DMF.

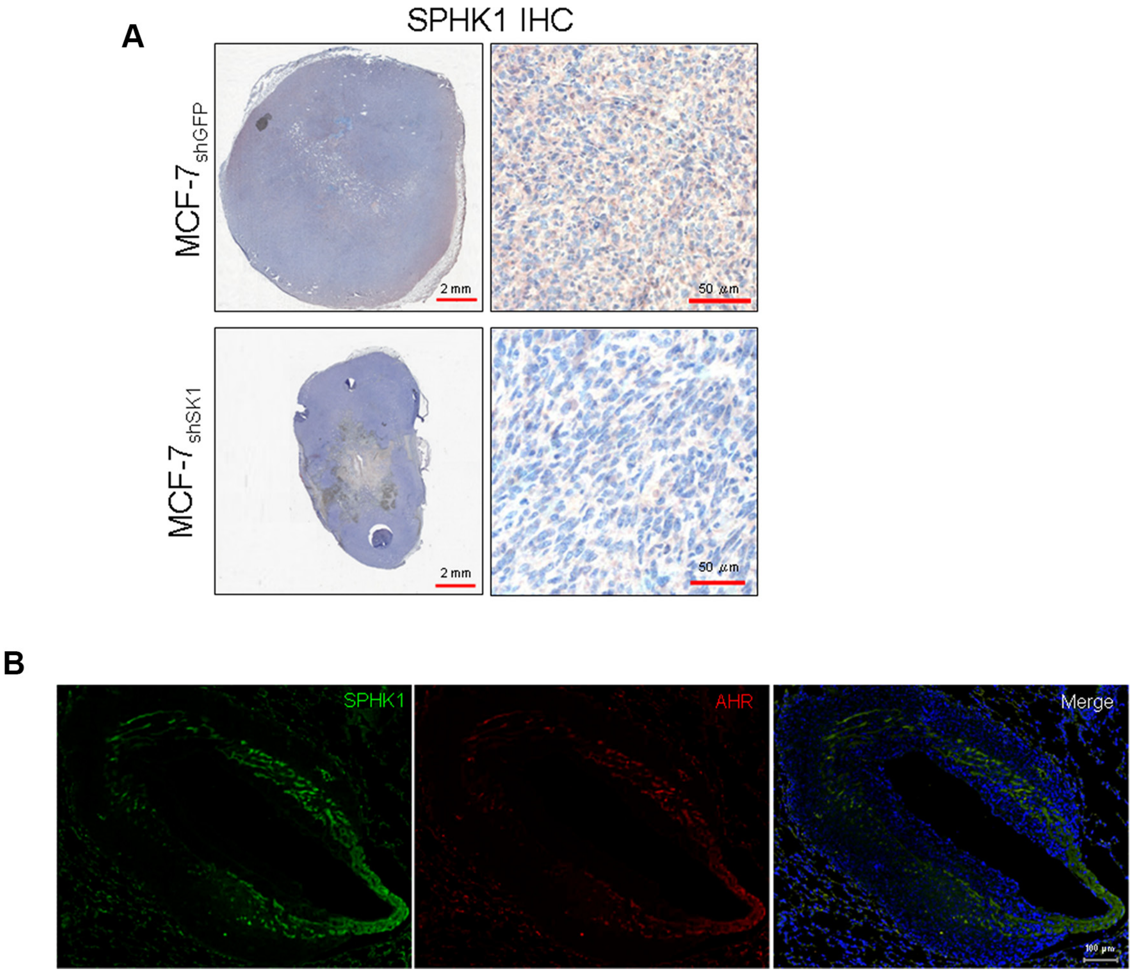


**Supplementary Figure S2:** (**A**) IHC analysis for SPHK1-expressing cells in tumor tissue sections of mice transplanted with MCF-$7_{shSPHK1}$ or MCF-$7_{shGFP}$ cells. (**B**) Immunofluorescence analysis of lung metastatic nodule of MCF-$7_{shGFP}$ cells as indicated to detect SPHK1 (green) and AHR (red). Nuclei were stained with DAPI (blue), and yellow color indicates SPHK1 and AHR colocalization.

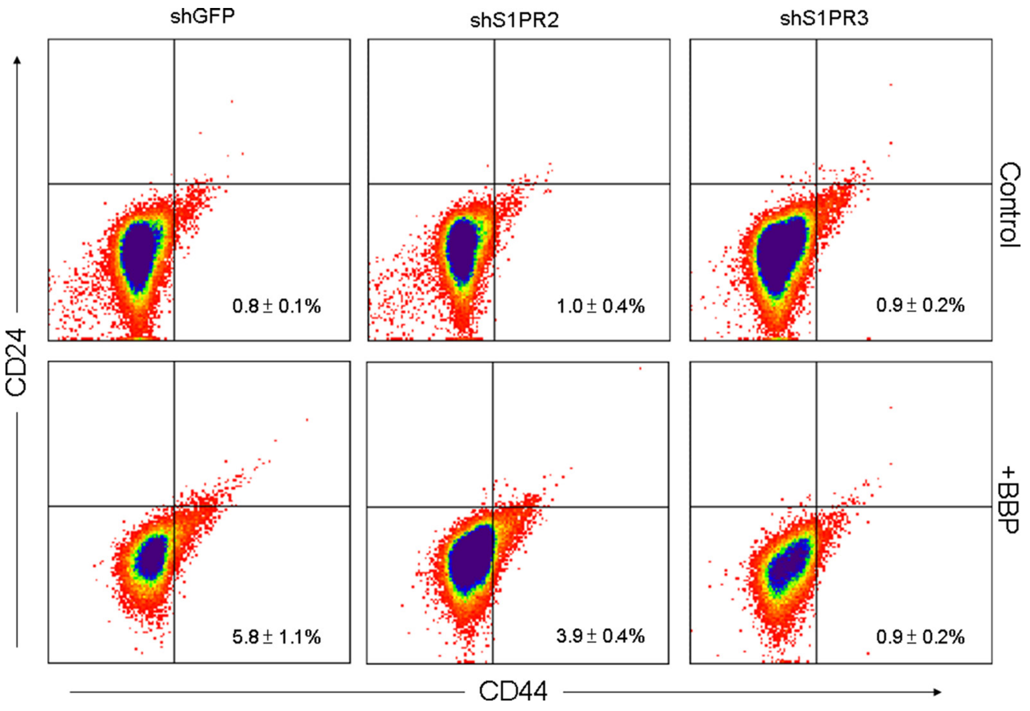


**Supplementary Figure S3: Flow cytometry analysis of surface staining of MCF-7 cells for CD24 and CD44.** Cells treated BBP for 24 hours after transfection with control, S1PR2 or S1PR3 shRNAs overnight. Gates are based on isotype controls.

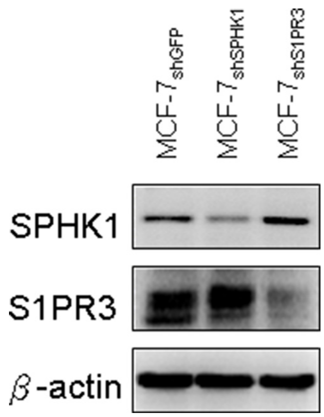


**Supplementary Figure S4: Representative immunoblots of SPHK1 and S1PR3 in the lysates of MCF-7 cells transfected with control, SPHK1 or S1PR3 shRNA.** β-actin was used as a loading control.
